# Increasing Resource Parents’ Sensitivity towards Child Posttraumatic Stress Symptoms: a Descriptive Study on a Trauma-Informed Resource Parent Training

**DOI:** 10.1007/s40653-017-0162-z

**Published:** 2017-06-24

**Authors:** Maj R. Gigengack, Irma M. Hein, Robert Lindeboom, Ramón J. L. Lindauer

**Affiliations:** 1 Department of Child and Adolescent Psychiatry, Academic Medical Center, University of Amsterdam, Meibergdreef 5, 1105 AZ Amsterdam, the Netherlands; 2 de Bascule, Academic Center for Child and Adolescent Psychiatry, Meibergdreef 5, 1105 AZ Amsterdam, the Netherlands; 3 Department of Clinical Epidemiology, Biostatistics and Bioinformatics, Master Evidence based practice in Health Care, Academic Medical Center, University of Amsterdam, Amsterdam, the Netherlands

**Keywords:** Posttraumatic stress disorder, Resource parents, Foster care, PTSD recognition, Upbringing stress, Child PTSD, Resource parent curriculum, Training

## Abstract

Resource parents are often insufficiently prepared for recognizing and managing posttraumatic stress symptoms (PTSS) in their traumatized foster children, which can put a successful foster placement at risk. The Resource Parent Curriculum (RPC) developed by the National Child Traumatic Stress Network is designed to increase resource parents’ sensitivity towards child PTSS. This study explores the effect of the RPC on resource parents’ recognition of child PTSS, resource parents’ perceived upbringing stress in caring for their foster child, and child PTSS before entering the RPC (T0), after completing the RPC (T1) and at six-month follow-up (T2). Results (*n* = 108) show an increase in recognition of child PTSS and a decrease in resource parents’ experienced upbringing stress and child PTSS over time. Findings suggest that the RPC increases resource parents’ trauma sensitivity. However, child PTSS severity remains high. To address foster children’s PTSS, child trauma-focused treatment appears needed in addition to the RPC.

## Background

Internationally, a large amount of children currently grow up in foster care: 402,378 in the United States, and 18,175 in the Netherlands (Child Welfare Information Gateway, 2015; Pleegzorg Nederland 2015). Many of them have experienced persistent and multiple child maltreatment. In particular neglect, physical abuse and sexual abuse are common reasons for placement in foster care (Oswald et al. 2010). Mental health problems that frequently arise in maltreated children include behavioral and emotional disorders (Burns et al. 2004), posttraumatic stress disorder (PTSD; Dubner and Motta 1999) and attachment disorder (Zeanah et al. 2004), which may lead to problems in daily functioning in the foster family, at school and in relationships.

Parenting foster children can be challenging for foster parents, especially because of the mental health problems many foster children are facing (Chamberlain et al. 2006; Sullivan et al. 2016). Farmer et al. (2005) showed that multiple conduct problems, hyperactivity and violent behavior in foster children are significantly related with foster parents’ experienced strain during the placement. Foster parents may lack the awareness that these foster children’s mental health problems result from chronic traumatization and are often insufficiently prepared for providing adequate care for traumatized children (Sullivan et al. 2016). As a result, this lack of trauma-informed perspective can put a successful foster placement at risk (Sullivan et al. 2016). Previous studies reported that higher levels of behavior problems and depression in foster children predict placement disruption (Barth et al. 2007; Chamberlain et al. 2006; Oosterman et al. 2007). In turn, placement disruptions may negatively affect a child’s development and aggravate existing mental health problems (Sullivan et al. 2016).

In order to reduce disruption of foster placement, it is vital to supply foster, therapeutic, adoptive, and kinship parents (hereby referred to as resource parents) with necessary knowledge and skills for providing adequate care for chronically traumatized children. For this goal “Caring for Children Who Have Experienced Trauma: A Workshop for Resource Parents”, often referred to as the Resource Parent Curriculum (RPC), was developed by over 30 experts at the National Child Traumatic Stress Network (NCTSN; Grillo et al. 2010a). This curriculum is uniquely designed to help resource parents provide a trauma-informed response to their foster or adopted child’s behaviors. Through an increased understanding of how traumatic events can affect a child’s development, resource parents can help their foster or adopted children heal from the effects of trauma and form healthy attachments. The curriculum was translated into Dutch by Coppens and Van Kregten (2012) and implemented in multiple institutions for child mental health in the Netherlands.

An initial evaluation of the RPC in the United States by Sullivan et al. (2016) showed indications that the RPC is effective in increasing resource parents’ knowledge about trauma-informed parenting and improving their skills for providing care for traumatized children. Recent file studies in the Netherlands demonstrated that resource parents who participate in the RPC report a high satisfaction and an increase in scores on training goals (Coppens and Van Kregten 2015; Lindauer et al. 2013). The Dutch studies conducted so far used the evaluation tool that was provided by the curriculum, which assesses the goals of the program on a 10-point scale. However, according a literature review, research in this area with standardized instruments is lacking in the Netherlands. Furthermore, to our knowledge the current study in the Netherlands is one of the first to evaluate the RPC outside the USA where the RPC was developed.

The aim of the present study is to evaluate the effect of the RPC on resource parents’ sensitivity towards child posttraumatic stress symptoms and on their perceived upbringing stress. First, since the RPC aims at increasing resource parents’ knowledge of PTSS, we hypothesize that resource parents recognize more child posttraumatic stress symptoms after completion of the RPC than before. Second, because the RPC focuses on increasing resource parents’ awareness that the child’s mental health problems result from chronic traumatization and on improving trauma-informed parenting skills, we expect a reduction in resource parents’ perceived upbringing stress. With perceived upbringing stress we refer to the upbringing stress resource parents experience in caring for their foster child. Besides stress that comes with upbringing in general, resource parents’ perceived upbringing stress could be influenced by factors unique to foster care, like difficulties in contact with the foster children’s biological family or foster children’s mental health problems (i.e. conduct problems, hyperactivity or violent behavior; Farmer et al. 2005). If resource parents understand and are able to manage the child’s mental health symptoms, this is hypothesized to lead to a decrease of resource parents’ perceived upbringing stress. Third, we explore the potential secondary effect of the RPC on the child’s PTSS. We hypothesize a possible decrease in child PTSS as a result of an improved interaction between resource parents and their foster child after the RPC. Finally, we evaluate resource parents’ satisfaction with the RPC. In accordance with previous studies (Coppens and Van Kregten 2015; Lindauer et al. 2013; Sullivan et al. 2016), we hypothesize that resource parents will report high satisfaction rates with the content of the RPC and the usefulness of the RPC in daily life.

## Method

### Participants and Procedure

From January 1st, 2013 until February 1st, 2015 resource parents participating in the RPC were prospectively enrolled. All resource parents and their foster or adopted children (hereby referred to as foster children) were referred to a specialized outpatient clinic for child and adolescent psychiatry, de Bascule, in Amsterdam, the Netherlands. All foster children were referred to the Center of Trauma and Family of de Bascule, a subdivision that provides treatment for foster families and (chronically) traumatized children. All participating foster children were under the age of 18 and experienced one or more traumatic events. The RPC is part of the standard treatment program offered to all resource parents who are referred to de Bascule.

A total of 112 resource parents participated in the RPC during the study period and were eligible for study participation, of whom 108 were included, four resource parents elected not to participate. In 34 cases, two resource parents of one foster/adopted child participated, resulting in data on 91 foster children and 93 resource families (two foster children lived in two different resource families). Data from these 34 resource parents were included in the analyses, because we aimed to explore the effect of the RPC on the sensitivity and skills of resource parents individually (not on families). Baseline characteristics are shown in Table 1.

**Table 1:** Baseline characteristics of resource parents (*n* = 108), foster/adopted children (*n* = 91) and resource families (*n* = 93)

	*n* (%)	Mean (*SD*, min-max)
Gender resource parents		
Male	32 (29.6)	-
Female	76 (70.4)	-
Gender foster/adopted children		
Male	53 (58.2)	-
Female	38 (41.8)	-
Age foster/adopted children	-	9.8 (3.4, 4–17)
Type family		
Adoptive family	5 (5.4)	-
Foster family	65 (69.9)	-
Professional foster family	23 (24.7)	-
Intended duration of placement		
Short-term (≤ 1 year)	20 (21.5)	-
Long-term (placement until adulthood)	55 (59.1)	-
N/A (adoption)	5 (5.4)	-
Not reported	13 (14.0)	-
Kinship care^a^		
No	74 (79.6)	-
Yes	9 (9.7)	-
Not reported	10 (10.8)	-
Actual duration of placement (months)	-	31.2 (32.4, 0–151)
Number of children in resource family	-	4.0 (1.7, 2–8)
Biological children	-	2.0 (1.0, 1–4)
Foster/adopted children	-	2.0 (1.5, 1–7)
Experience as a resource parent		
0–3 years	42 (45.2)	-
3–6 years	23 (24.7)	-
6–10 years	11 (11.8)	-
> 10 years	12 (12.9)	-
Not reported	5 (5.4)	-

^a^Kinship care: Care for foster/adopted children by a member of the child’s social network (e.g. a relative, neighbor or family friend)

As part of the standard protocol, resource parents filled out questionnaires at three different times. First, these were filled out before entering the RPC (T0), and second, after completing the eight-session RPC. Resource parents filled out questionnaires immediately after completing the last session of the RPC (T1). Third, and finally, the questionnaires were filled out at six-month follow-up (T2). Not all resource parents completed all measurements. Of the 108 participating resource parents, 102 resource parents filled out the questionnaires at T0 (5 parents were not present at T0 and started the RPC after the first session, 1 parent did not complete the measures). At T1, 91 resource parents completed all measures (12 parents were lost to follow up, 1 parent stopped the RPC, 3 parents filled out questionnaires about another child, 1 parent stopped the foster placement). At T2, 56 resource parents filled out the questionnaires (41 parents were lost to follow up, 2 parents stopped the RPC, 1 parent filled out questionnaires about another child, 7 parents stopped the foster placement and 1 parent could not be traced).

At T0 resource parents were informed about the study by the RPC trainers and were asked permission to use the questionnaire data for the study. Informed consent was obtained from the participating resource parents. The medical ethics committee of the Academic Medical Center approved the study. All procedures performed in this study were in accordance with the 1964 Helsinki declaration and its later amendments or comparable ethical standards.

### Intervention

The RPC consists of the following eight modules: 1) “*Introductions*”, 2) “*Trauma 101*” (types of trauma), 3) “*Understanding the effect of trauma*”, 4) “*Building a safe place*”, 5) “*Dealing with feelings and behaviors*”, 6) “*Connections and healing*”, 7) “*Becoming an advocate*” and 8) “*Taking care of yourself*” (Grillo et al. 2010b). Each module covers one or more topics aimed at improving resource parents’ knowledge and skills for providing adequate care for traumatized children. The topics are shown in Table 2 (also available on the website: http://nctsn.org/products/caring-for-children-who-have-experienced-trauma, National Center for Child Traumatic Stress 2010).

**Table 2:** Modules and topics of the Resource Parent Curriculum (Grillo et al. 2010b)

Module	Topics
1: Introductions	Introducing the RPC Introducing the concept of trauma-informed parenting
2: Trauma 101	Define child trauma / types of child trauma Children’s response to trauma PTSD Resilience
3: Understanding trauma’s effect	Effect of trauma on children’s development Responses to trauma in children of different ages
4: Building a safe place	Promote safety for traumatized children Trauma reminders (intrusion)
5: Dealing with feelings and behaviors	Feelings and emotions of traumatized children (understanding and managing) Acting out behavior of traumatized children (understanding and modification)
6: Connections and healing	Influence of trauma on children’s perspective on themselves and the future Traumatized children’s connections and relationships
7: Becoming an advocate	Help others to understand child traumatic stress and its impact Promote and support trauma-focused treatment for the child
8: Taking care of yourself	Awareness of own health Recognition of warning signs

The RPC was provided in eight bi-weekly sessions of 2.5 h by two trained health care professionals and attended by 10 to 15 resource parents each training. One follow-up session of 2.5 h was given six months after the last session.

### Measures

Baseline characteristics of the foster children (e.g. age and gender), the resource parents (e.g. gender and years of experience as a resource parent) and the resource families (e.g. type of family and number of children living in the resource family) were measured by a questionnaire on demographic information filled out by the resource parents.

Parental stress was measured by the Burden of Upbringing Questionnaire (Opvoedingsbelastingvragenlijst (OBVL); Vermulst et al. 2011) filled out by the resource parents. This questionnaire consists of 34 items divided over five subscales: (1) “*Problems in the educator-child relationship*”, (2) “*Upbringing problems*”, (3) “*Depressive moods*”, (4) “*Restricted role*” and (5) “*Physical health*”. The total score is categorized into five groups, ranging from “*No problems*” to “*Very serious problems*”. Overall reliability and validity of the Burden of Upbringing Questionnaire are good (Vermulst et al. 2015). The overall internal consistency, which was estimated with Cronbach’s alpha and McDonald’s omega, was good: Cronbach’s alpha ranged from .74 to .87 for the subscales and from .89 to .91 for the total score, McDonald’s omega ranged from .87 to .98 for the subscales and from .96 to .97 for the total score (Vermulst et al. 2015).

Child PTSS was measured by the Children’s Revised Impact of Event Scale (CRIES) parental version (Verlinden et al. 2005). The CRIES is based on PTSD symptoms according to the Diagnostic and Statistical Manual of Mental Disorders, Fourth Edition, Text Revision (DSM-IV-TR). The CRIES offers a total score (0–65) and three subscale scores on the symptom clusters intrusion (0–20), avoidance (0–20) and hyperarousal (0–25). Initial psychometric properties of the CRIES parental version showed good reliability and validity (Verlinden et al. 2014a). The internal consistency was *α* = 0.87 for the total score, 0.78 for the intrusion cluster, 0.78 for the avoidance cluster and 0.71 for the hyperarousal cluster. Calculation of the convergent validity showed a strong correlation between the CRIES total score and the total score of another PTSD questionnaire, the Trauma Symptom Checklist for Young Children (TSCYC) (*r* = .73, *p* < .001). Furthermore, calculation of the divergent validity showed moderate correlations between the CRIES total score and three subscales and the total behavioral problem scale of the Strengths and Difficulties Questionnaire (SDQ; total score *r* = .38, *p* = .005; intrusion *r* = .37, *p* = .008; avoidance *r* = .15, *p* = .300 and hyperarousal *r* = .46, *p* = .001; Verlinden et al. 2014a). If resource parents did not know the answer to a CRIES item they were asked to fill out a question mark. The number of question marks was used as an indicator of the resource parents’ recognition of posttraumatic stress symptoms in their foster child. Although this measurement merely represents an indication of parents’ PTSS recognition, it was used, as no validated test is currently available. In accordance with previous studies, the missing items were scored 0 for calculation of the total score. If two or more items of a subscale were not filled out, a total score could not be calculated (Verlinden et al. 2014b). Cut-off score of the CRIES parental version is 31 (Verlinden et al. 2014a).

Participants’ satisfaction with the RPC was measured by an evaluation questionnaire. Parents rated the 8 modules and the overall usefulness of the RPC in daily life on a 0 to 5 response scale (0 = not satisfied, 3 = neutral, and 5 = very satisfied).

### Data Analysis

Baseline characteristics and participants’ satisfaction with the RPC were summarized with mean ± SD and range for continuous variables and numbers and percentages for categorical variables. Attrition analysis was conducted in order to determine differences between responders and non-responders on demographic characteristics, number of question marks filled out on the CRIES at baseline, baseline OBVL scores and baseline CRIES scores. For the analysis and significance testing of the repeated measures over time, we used linear mixed models (LMM; West et al. 2015). To test the changes over time on the different outcome measures, time was entered as a fixed effect. Using LMM we could enter all available measurements; even if a participant did not complete a specific measurement, the data of the other measurements could still be included in the analysis (PTSS recognition *n* = 108, OBVL *n* = 105, CRIES *n* = 101). Magnitude of the differences between mean scores on the time points were examined by calculating effect sizes using Cohen’s *d* ((Mean_1_ - Mean_2_) divided by the pooled *SD*, where pooled *SD* = √[(*SD*_1_^2^+ *SD*_2_^2^)] / 2). Effect sizes were interpreted according to Cohen’s classification: < 0.20 small, around 0.50 medium and >0.80 large (Cohen 1992). The internal consistency of the CRIES and the OBVL at the three different time points was estimated with Cronbach’s alpha. We used IBM Statistical Product and Service Solutions (SPSS) 19 for all analyses.

## Results

Attrition analysis showed that responders and non-responders only differed on placement type: adoptive families were more likely to complete all three measurements than (professional) foster families (χ^2^ = 8.3, *p* = .02). No significant differences were found between responders and non-responders in terms of resource parents gender, foster child gender, foster child age, actual and intended duration of placement, kinship foster care/adoption, number of children living in the foster/adoptive family (biological, foster/adoptive, total), or number of years as a foster/adoptive parent. Moreover, no significant differences were found at baseline between responders and non-responders on number of question marks filled out on the CRIES (*t*(98) = 0.56, *p* = .58), OBVL scores (*t*(94) = 0.98, *p* = .33) and CRIES scores (*t*(63) = 0.88, *p* = .38).

Magnitude of the changes on the PTSS recognition, CRIES scores and OBVL scores between time points are shown in Table 3. The number of question marks filled out on the CRIES significantly decreased between T0 and T1 and between T0 and T2. Moderately large effect sizes were seen for these two time points (respectively 0.74 and 0.72). OBVL scores significantly decreased between T0 and T2, the effect size was small (0.07). Furthermore, total CRIES scores significantly decreased between T0 and T1 and between T0 and T2, with small to medium effect sizes (respectively 0.38 and 0.40). The reliability of the measures at the different time points ranged from *α* = 0.79 to *α* = 0.86 for the CRIES total score and from *α* = 0.93 to *α* = 0.94 for the OBVL total score.

**Table 3:** Differences between Measurements at Three Time Points

	Baseline (T0)			Post test (T1)				Follow-up (T2)					Effect size *d*		
Measures	*n*	*M*	*SD*	*n*	*M*	*SD*	*p* (T0-T1)	*n*	*M*	*SD*	*p* (T0-T2)	*p* (T1-T2)	T0-T1	T0-T2	T1-T2
PTSS recognition (# of question marks)	100	1.57	2.38	89	0.25	0.88	< .001**	53	0.30	0.77	< .001**	1.00	0.74	0.72	−0.06
OBVL	96	62.89	9.92	91	62.35	10.49	1.00	55	62.20	10.33	.03*	.09	0.05	0.07	0.01
CRIES	65	34.08	12.34	82	29.12	13.54	.04*	49	28.67	14.33	.008**	.66	0.38	0.40	0.03

Effect size expressed in Cohen’s *d*

*p < 0.05, **p < 0.01 by linear mixed models

Participants’ satisfaction with the RPC was generally high. All modules had mean satisfaction scores exceeding 4, except for module 1 (Introductions) which showed a mean satisfaction score of 3.71. The mean satisfaction score for the overall usefulness of the RPC in daily life was 3.92.

## Discussion

The RPC appears to have a positive effect on resource parents’ recognition of child PTSS, which was represented by a decreased number of question marks resource parents filled out on the CRIES. This recognition is achieved after completing the curriculum and is still present at the six-month follow-up, suggesting that the positive effect on resource parents’ recognition of child PTSS remains stable over time. Targeting the lack of recognition in resource parents, the RPC appears to achieve the goal of improving sensitivity. This finding confirms our hypothesis and is in line with a previous study of Sullivan et al. (2016), who also found that resource parents were more knowledgeable about child PTSS after the RPC. Furthermore, in line with our second hypothesis, the upbringing stress experienced by resource parents decreased although the effect size is small. This decrease did not occur during the training, but in the follow-up period. Resource parents might need some time to digest their newly acquired knowledge and to implement their skills in the resource family.

In addition, resource parents reported significantly less PTSS in their child after the RPC and at follow-up than at the start of the training program. Resource parents’ increased trauma sensitivity might hypothetically have a positive effect on the parent-child interaction, which in turn might indirectly lead to a reduction in child PTSS. However, the effect size was moderate and child PTSS was still severe after the training. Therefore, the need for evidence based child trauma-focused treatment, like Eye Movement Desensitization and Reprocessing (EMDR) or Trauma-Focused Cognitive Behavioral Therapy (TF-CBT), remains unaltered. Finally, as hypothesized, participants’ satisfaction with RPC was generally high.

This descriptive study has some limitations that should be noted. First, although the design fits our aim to evaluate the effects of the RPC training in the Netherlands it has certain drawbacks. Despite the fact that the RPC appears to have a positive effect on resource parents’ recognition of child PTSS, on their perceived upbringing stress and on the children’s CRIES scores, the pre-post design offers no certainty on causality between the RPC and the outcome measures, only a randomized controlled design could accomplish this. Second, the number of resource parents who completed the measurements decreased over time, which lead to a smaller sample size at T2. However, attrition analysis demonstrated that completers and non-completers did not differ on all baseline measurements. Third, only resource parents referred to a specialized treatment center were included. The referral criteria of the treatment center include children with severe symptoms or comorbidity or children who did not respond to previous professional treatment. Although the treatment center serves a wide region (metropolitan and surroundings), the referral criteria might limit the generalizability of this study. Finally, we used number of question marks filled out by resource parents on the CRIES as an indicator of their PTSS recognition, because there are currently no validated measures available. Clearly the CRIES is not designed for this purpose, however this measure might be a valid proxy for PTSS recognition.

Future studies should include possible confounding factors, like number of foster placement breakdowns. We recommend future studies to include more targeted measures to assess changes in resource parents’ sensitivity towards child PTSS. Specifically, we suggest observational measures utilizing structured and free play situations in order to evaluate the interaction between the resource parent and foster child.

## Conclusion

The RPC developed by the NCTSN appears to provide for increasing resource parents’ recognition of child PTSS, and by that for some reduction in resource parents’ perceived upbringing stress and child PTSS reported by resource parents. This might have a positive effect on foster children’s placement, as previous studies show that resource parents’ perceived upbringing stress and foster children’s mental health problems increase the risk of placement disruption (Barth et al. 2007; Chamberlain et al. 2006; Farmer et al. 2005; Oosterman et al. 2007). Further research is needed to confirm this effect. We recommend long-term follow up studies on the effect of the RPC and foster children’s placement stability. Also, we suggest the use of more targeted measures to assess the effect of the RPC on resource parents’ trauma sensitivity.

The RPC appears to be a useful method to supply resource parents with the necessary knowledge and skills for providing adequate care for traumatized foster children. Besides, resource parents’ high satisfaction rates with the RPC implies that the content of the curriculum is suitable for resource parents’ and the provided knowledge and skills are applicable to resource parents’ daily life. Therefore, we recommend the implementation of the RPC in institutions of child mental health and foster care.

While foster children’s PTSS appears to decrease as an indirect consequence of resource parents’ increased trauma sensitivity, the severity of child PTSS remained high directly after the RPC and at six-month follow-up. In order to address foster children’s PTSS, we recommend trauma-focused treatment for foster children in addition to the RPC.
